# Frequency of EBV LMP-1 Promoter and Coding Variations in Burkitt Lymphoma Samples in Africa and South America and Peripheral Blood in Uganda

**DOI:** 10.3390/cancers10060177

**Published:** 2018-06-02

**Authors:** Hsiao-Mei Liao, Hebing Liu, Heiyan Lei, Bingjie Li, Pei-Ju Chin, Shien Tsai, Kishor Bhatia, Marina Gutierrez, Sidnei Epelman, Robert J. Biggar, Francis Nkrumah, Janet Neequaye, Martin D. Ogwang, Steven J. Reynolds, Shyh-Ching Lo, Sam M. Mbulaiteye

**Affiliations:** 1 Center for Biologics Evaluation and Research, Food and Drug Administration, White Oak, MD 20993, USA; hsiao-mei.liao@fda.hhs.gov (H.-M.L.); hebing.liu@fda.hhs.gov (H.Liu.); lei.haiyan@nih.gov (H.Lei.); bingjie.li@fda.hhs.gov (B.L.); pei-ju.chin@fda.hhs.gov (P.-J.C.) ; shien.tsai@gmail.com (S.T.); 2 Cancer Genetics, Inc., Rutherford, NJ 07070, USA; kishor.bhatia@cgix.com; 3 Laboratorio Stamboulian, Laboratorio Stamboulian, Buenos Aires 1414, Argentina; mgutierrez@stamboulian.com.ar; 4 Department of Pediatric Oncology, St Marcelina Hospital, Sao Paolo 08270-070, Brazil; epelman@uol.com.br; 5 Infections and Immunoepidemiology Branch, National Cancer Institute, Bethesda, MD 20892, USA; rjbiggar@gmail.com; 6 Noguchi Memorial Institute, Kor Le Bu University, P.O. Box LG 581 Legon, Accra, Ghana; FNkrumah@noguchi.ug.edu.gh; 7 Department of Child Health, University of Ghana, P.O. Box LG 25 Legon, Accra, Ghana; janet.neequaye@yahoo.com; 8 EMBLEM Study, St. Mary’s Hospital, Lacor, P.O. Box 180, Gulu, Uganda; ogwang.martin@lacorhospital.org; 9 Division of Intramural Research, National Institute of Allergy and Infectious Diseases, Bethesda, MD 20892, USA; sjr@jhmi.edu

**Keywords:** Epstein-Barr virus, Burkitt lymphoma, Africa, Latin America, next-generation sequencing, LMP-1

## Abstract

Epstein-Barr virus (EBV) is linked to several cancers, including endemic Burkitt lymphoma (eBL), but causal variants are unknown. We recently reported novel sequence variants in the LMP-1 gene and promoter in EBV genomes sequenced from 13 of 14 BL biopsies. Alignments of the novel sequence variants for 114 published EBV genomes, including 27 from BL cases, revealed four LMP-1 variant patterns, designated A to D. Pattern A variant was found in 48% of BL EBV genomes. Here, we used PCR-Sanger sequencing to evaluate 50 additional BL biopsies from Ghana, Brazil, and Argentina, and peripheral blood samples from 113 eBL cases and 115 controls in Uganda. Pattern A was found in 60.9% of 64 BL biopsies evaluated. Compared to PCR-negative subjects in Uganda, detection of Pattern A in peripheral blood was associated with eBL case status (odds ratio [OR] 31.7, 95% confidence interval: 6.8–149), controlling for relevant confounders. Variant Pattern A and Pattern D were associated with eBL case status, but with lower ORs (9.7 and 13.6, respectively). Our results support the hypothesis that EBV LMP-1 Pattern A may be associated with eBL, but it is not the sole associated variant. Further research is needed to replicate and elucidate our findings.

## 1. Introduction

Epstein-Barr virus (EBV) is a gammaherpesvirus that is causally associated with multiple cancers [1,2,3,4,5,6], including endemic Burkitt lymphoma (eBL), an aggressive pediatric cancer that occurs with high incidence in African countries with high malaria endemicity [7]. Although EBV infection establishes asymptomatic lifelong infection in >90% of the world’s population [8], EBV-related cancers develop in a small proportion of EBV infected people; this proportion resulted in an estimated burden of about 120,000 cancers in 2012 or about 5.5% of all infection-associated cancers [9]. The small proportion of EBV-infected people who progress to cancer could be due to the effects of high-risk variants with greater propensity for carcinogenicity [10]. The strong regional distributions of eBL in sub-Saharan Africa, and nasopharyngeal carcinoma (NPC) in the Far East China and parts of North African, stand in marked contrast to the ubiquitous distribution of EBV [11]. These regional distributions may be interpreted as epidemiological clues about the regional distribution of high-risk EBV tumor-specific variants that play a role in the pathogenesis of eBL and NPC [10,12].

The nature of high-risk EBV variants is currently unknown. EBV sequence diversity in EBNA-3A, EBNA-3B, and EBNA-3C genes [13] enables EBV to be grouped into two major types (1 and 2, also known as A or B), but it is unrelated to carcinogenicity [10]. EBV type 2 appears to be over-represented in samples collected in Africa [14], but recent studies conducted in the United Kingdom have reported type 2 EBV in healthy people native to that region [15]. Studies of EBV diversity in the EBV nuclear antigen 1 (EBNA-1), which is expressed in EBV-positive eBL, or transcription factor Zta (BZLF-1), and latent membrane protein 1 (LMP-1), which have transforming properties [16], have not successfully demonstrated an association of diversity in those genes with risk of EBV related cancers [10]. The development of whole-genome high throughput sequencing (HTS) methods and their application to EBV provides new opportunities for the discovery of EBV genetic variants that may be associated with the risk of EBV-related cancers [17]. 

We recently reported novel sequence variants in the LMP-1 gene and promoter in EBV genomes sequenced by HTS from 13 of 14 BL biopsies from Ghana, Brazil, and Argentina [18]. Alignment of the novel sequences for 114 published EBV genomes, including 27 from BL cases, revealed four LMP-1 patterns, which we designated as Patterns A to D (as shown in Table 1) [18]. Pattern A was characterized by 11 SNVs located in the LMP-1 promoter and the flanking LMP-2B noncoding region, and 12 SNVs located in exons 1 and 3 of the LMP-1 gene that were associated with nine amino acid changes (Table 1). Pattern B was characterized by 28 SNVs located in the promoter, and one SNV located in exon 1 and associated with an amino acid change. Pattern C was characterized by two SNVs located in the promoter, and one SNV located in exon 1 at position 13 (that is also found in Pattern A) that is associated with an amino acid change. Pattern D was the unaltered sequence in the EBV reference genome NC_007605 (wild-type EBV). Interestingly, Pattern A was more enriched in the EBV genomes sequenced from BL tumors than EBV genomes sequenced from non-BL samples (51.8% versus 7%) in the 114 EBV genomes, leading us to hypothesize that Pattern A is associated with BL [18]. Confirmation of this hypothesis could present promising new strategies for EBV vaccines or targets for high-risk EBV variants [19].

**Figure 1:**
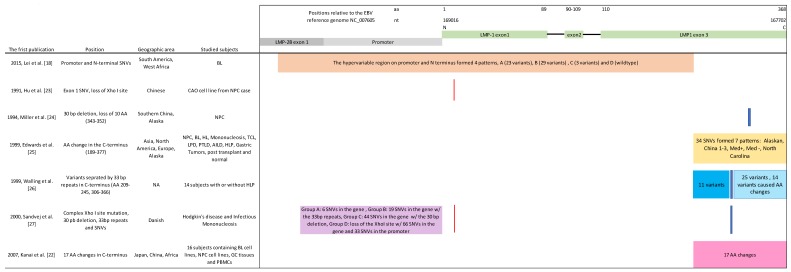
A schematic representation of the LMP-1 promoter and coding region showing the relative positions of the variations in LMP-1 described and used in different studies to classify EBV LMP-1 genetic diversity (See reference number for first publication). Color shadows highlight the investigated regions or positions of each publication. Different color shades are used when the specific variations identified in the studied regions are different. Use of the same color shade at the same region, e.g., for variations in Hu et al [18] and Sandvej et al [27] indicate the same exact variation used in the two studies. The relative positions of the variations correspond to the illustration of the LMP-1 gene on the top.

This study was conducted: (a) to use EBV genome-wide data [15,18] to classify LMP-1 genetic diversity according the Lei LMP-1 Patterns and according to six other published LMP-1 variant classifications (as shown in Figure 1 and Table 2), and determine whether these classifications are independent; (b) to increase the sample size of BL biopsies studied to investigate the enrichment of Pattern A LMP-1 variant in BL biopsies by evaluating 50 additional samples from Ghana, Brazil, and Argentina using PCR-Sanger sequencing; and (c) to assess the distribution of the Pattern A LMP-1 variant in peripheral blood samples of 113 eBL cases and 115 controls enrolled from the same geographical area in the Ugandan component of the Epidemiology of Burkitt’s Lymphoma in East-African Children and Minors (EMBLEM) study [20,21].

## 2. Results

### 2.1. Comparison of EBV LMP-1 Genetic Diversity in 114 Published Genomes Classified by Seven Techniques

Figure 1 shows the regions of genetic variation in the LMP-1 gene and promoter described by Lei et al. [18] and 6 other investigators [22,23,24,25,26,27]. Briefly, genetic variation was reported in the coding region of the gene but not the promoter by five of seven reports Hu et al. [23], Miller et al. [24], Edwards et al. [25], Walling et al. [26], and Kanai et al. [22]). Genetic variation was reported in both the LMP-1 gene and promoter by two studies (Sandvej et al. [27], and Lei et al. [18]). Furthermore, genetic variations were identified in the LMP-1 exon 1 in three reports (Sandvej et al. [27], Lei et al. [18], and Hu et al. [23]) and in the LMP-1 exon 3 for all reports except that of Hu et al. [23]. As shown in Figure 1, the variations reported by Lei et al. [18] are physically independent from variations reported by the six other reports. Genetic variations involving the 30 bp deletion and 33 bp repeats in LMP-1 exon 3 were used to classify LMP-1 genetic diversity by three reports (Miller et al. [24], Wallings et al. [26], and Sandvej et al. [27]).

Table 2 summarizes the LMP-1 genetic classifications based on Lei et al. [18], Hu et al. [23], Miller et al. [24], Walling et al. [26], and Sandvej et al. [27] for the 114 EBV genomes previously studied in Lei et al. [18]. The results are sorted by Lei patterns A to D [18] and disease phenotype BL, NPC, Hodgkin lymphoma (HL), post-transplant lymphoproliferative disease (PTLD), gastric cancer (GC), and healthy samples (lymphoblastoid cell line [LCL], Spontaneous transformed lymphoblastoid cell line [sLCL], and Saliva). We note that 100% of the HTS data could be used to define LMP-1 genetic diversity according to Lei et al. [18], and that it was possible for 99.1% for classification by Miller et al. [24], for 97.4% for Hu et al. [23], and 96.5% in Edwards et al. [25]. However, HTS data could be used to classify only 6.1% of the samples according to Sandvej et al. [27] because Sandvej uses the number of 30 bp and 33 bp deletions to classify LMP-1 genetic variants, but the number of deletions cannot be accurately determined from HTS data.

**Figure 2:**
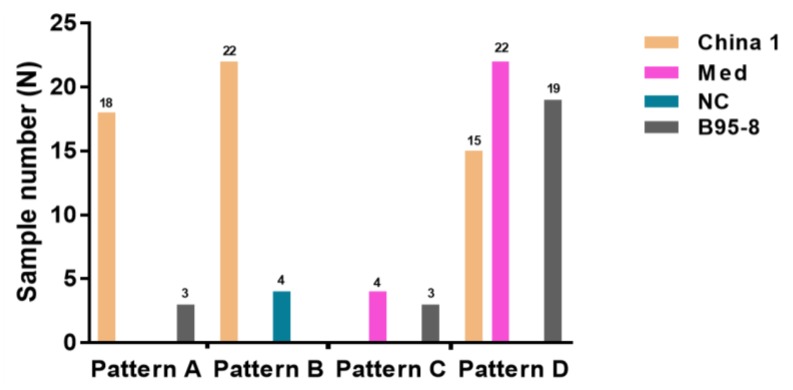
The cross classification of the 114 samples using LMP-1 Patterns according to Lei et al. [18] and the Edwards et al. [25] Med: Mediterranean, NC: North Carolina.

Figure 2 shows a pairwise classification of LMP-1 genetic diversity according to Edwards et al. [25] and Lei et al. [18] for 110 EBV genomes satisfactorily analyzed for both. The Edward’s system results in seven LMP-1 strains based on the nonsynonymous nucleotide changes in the C-terminus of LMP-1, and four EBV strains in the 110 analyzed EBV genomes: China 1, Mediterranean, North Carolina, and B95-8. The China 2, China 3, and Alaskan strains were not found in this set. As shown in Figure 2, the four Edward’s strains are not correlated with the Lei patterns, as indicated by the observation that different strains defined by one method can be identified in another strain using the other method, and vice versa. For example, China 1 Edward strains were found in Lei Patterns A, B, and D strains; the B95-8 Edward strain was found in Lei Patterns A, C, and D (Figure 2). A possible exception was the North Carolina (NC) Edwards strain, which only co-occurred with Lei Pattern B, which has been seen predominantly in the samples from the Far East. However, whether the North Carolina strain is correlated with Far East ancestry, as seems to be the case for Pattern B, is unclear, because some of the samples which were jointly classified as North Carolina and Pattern B were collected in Australia and Germany, and their ancestry is unknown to us. Because we have previously shown that the Lei Patterns A to D identify unique genetic variants that cluster together in phylogenetic trees based on the entire EBV genome sequence, on the sequence from EBNA1, or LMP-1 genes (Figure 1 in [18]). We conclude that LMP-1 genetic diversity according to Edward et al. [25] is independent of that described by Lei et al. [18], and may be less discriminatory for disease or geographical patterns.

**Figure 3:**
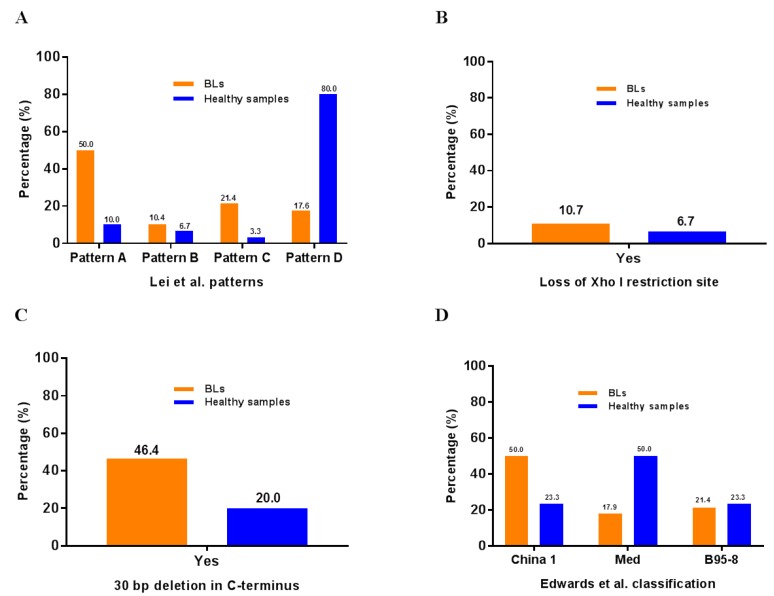
Different LMP-1 classifications applied to BL and healthy samples analyzed in the study of Lei et al. (2015) [18]. (**A**) The Patten A–D of Lei et al. [18] study. (**B**) The loss of Xho I site used in the study of Hu et al. [23] (**C**) 30 bp deletion in LMP-1 C terminus used in the study of Miller et al. [24] (**D**) The classification of the LMP-1 C-terminal variants used in the study of Edwards et al. [25].

Figure 3A–D shows the distribution of Lei Patterns A–D, Hu variants based on Xho I loss of restriction site, Miller variants based on 30 bp deletion in C-terminus, and Edward’s (defined in Table 2) in 28 BL EBV genomes and 30 lymphoblastoid cell lines and saliva EBV genomes from healthy subjects that were classified using HTS data. Enrichment of Pattern A was observed in BL EBV genomes, but not in EBV genomes from healthy samples (50.0% versus 10.0%, Figure 3A). Pattern D predominated in EBV genomes from healthy samples, and was observed relatively infrequently in BL EBV genomes (80.0% versus 17.6%, Figure 3A). The distribution of loss of the Xho I restriction site was rare both in EBV in BL samples and healthy subjects (10.7% versus 6.7%, Figure 3B). The 30 bp deletion in the C-terminus in exon 3 [24] was enriched in BL EBV genomes, and was relatively infrequent in EBV genomes from healthy subjects (46.4% versus 20.0%, Figure 3C). The frequency of China 1 strains was enriched in BL EBV genomes compared to EBV from healthy samples (50.0% versus 23.3%, Figure 3D), while the opposite pattern was observed for the Mediterranean strain; no differences were observed for B95-8 Edward’s strains. 

**Figure 4:**
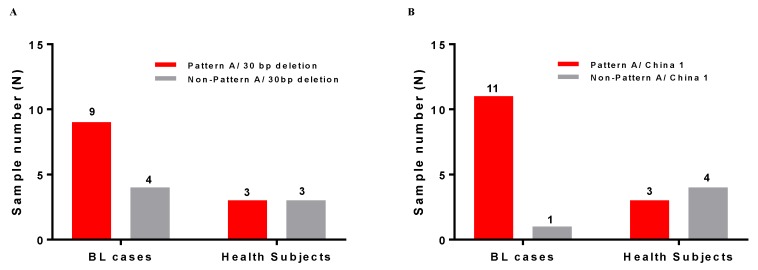
Figure shows co-distribution of Pattern A with the LMP-1 30 bp deletion (Panel **A**) and the China 1 variant (Panel **B**) in published EBV genomes obtained from 28 BL cases and 30 healthy subjects.

Given our findings above showing a preponderance of China 1 and 30 bp deletions in BL cases, the BL cases were jointly classified by Lei LMP-1 patterns and Edward’s China 1 strain or Lei patterns by Miller’s 30 bp deletion (Figure 4). We noted that the higher frequency of the 30 bp deletion (Figure 3C) or China 1 (Figure 3D) in BL EBV genomes was mostly explained by correlation with Lei LMP-1 Pattern A (Figure 4A, B). The North Carolina strain was not seen in the analyzed BL EBV genomes. This is not surprising, given our finding that North Carolina Edward’s strains are correlated with Pattern B (Figure 2), which is rarely seen in BL samples. The one BL sample that was classified as Pattern B in the Lei system was indeterminate on Edward’s classification (Table 2).

To confirm the enrichment of LMP-1 Pattern A in BL samples in a separate dataset, we aligned LMP-1 sequences of EBV genomes from 23 BL samples and 59 EBV genomes from healthy individuals published by Correia et al. [15], that did not overlap with samples analyzed in Lei et al. [18]. LMP-1 Pattern A variants were observed in four (17.4%) BL EBV genomes but in none of the 59 EBV genomes from healthy subjects (Table S5). Although this set is also somewhat limited by the lack of BL-free controls from the same cities and towns as the BL cases, the results are in the same direction as those comprising the original analysis [18]. The paucity of samples from healthy subjects, collected from the same cities and towns from which the BL samples were obtained, is a major limitation for any analysis based on the rapidly accumulating EBV HTS data. For example, while 20 BL samples in the data by Correia et al. [15] are from Africa, only four samples from healthy people are from Africa, and not from the same cities of towns at that (Table S6). Future collections are needed to address this disparity in sample inclusion, and more efforts must be made to enroll healthy people from the same cities and towns where the BL samples were collected, to increase the value of the generated data for investigations of associations between EBV genetic diversity and BL. 

### 2.2. Discovery of Novel Pattern A-Like Variations in Samples Studied by PCR-Sanger Sequencing

Sequence analysis of LMP-1 sequences from the 50 additional BL samples revealed single nucleotide variations, suggesting a shift from Pattern D to Pattern A, but not involving all the 23 Pattern A-defining variations (see Table 1). We designated these A-like variations “variant Pattern A”, and grouped them into four sub-patterns (Table 3). One variant A sub-pattern was characterized by 11 or 12 SNVs also observed in Pattern A, including one or two SNVs at positions 11 or 12 (Table 1 for position numbers) in the LMP-1 promoter, and 10 SNVs located at positions 13–23 in exon 1 and 3 of the N-terminus of LMP-1 gene (representative sample BLS000207, Table 3). This variant is designated the “Mid-length A-like Pattern (ML-A)”, because the relative location of SNVs are in the mid-region of Pattern A variations. The second and third variant A sub-patterns were characterized by 4 or 3 nt variations. The 3 nt variant Pattern A was characterized by 3 SNVs at positions 13–15 in exon 1 in the N-terminus of LMP-1 gene (see Table 1), while the 4 nt variant A has an SNV in the LMP-1 promoter at position 10 (representative samples BLS000292 and BLS000020, Table 3). A novel variation S24A was discovered in the samples with 3 or 4 nt variant A sub-patterns; this variation was not previously observed in Lei et al. [18] and is not part of the Lei classification system. The fourth variant A sub-pattern was characterized by 16 SNVs: seven were at positions 6–12 in the LMP-1 promoter, two were at positions 14 and 15 in exon 1, one was at position 23 in exon 3, and five were at positions 1–5 in LMP-2B exon 1 (representative sample BLS001943, Table 3). This sub-pattern was designated as “Partial A variant sub-pattern”, because it has the most SNVs observed in Pattern A. Our finding of novel variant Pattern A sub-patterns suggests that the EBV genetic diversity in the LMP-1 gene is more extensive than is currently known.

### 2.3. Frequency of Pattern A in 64 BL Tumors from Ghana, Argentina, and Brazil

Table 4 shows the results of 71 samples analyzed by PCR and Sanger sequencing from Argentina, Brazil, and Ghana, including 14 that were previously analyzed by HTS and validated by PCR and Sanger sequencing (the genotype of each sample is shown in Tables S2 and S3) [18]. Seven BL biopsies were EBV negative and excluded from frequency analysis. The distribution of Lei LMP-1 Patterns of the BL biopsies was A in 39 (60.9%) and D in 14 (21.9%) BL biopsies. Pattern B and C were observed in 1 (1.6%) biopsy each. Variant Pattern A of the Mid-Length type was observed in 7 (10.9%) BL biopsies. Analysis of variation by country shows Pattern A varying from 55% in Ghana to 80% in Brazil.

Two (3.1%) BL biopsies, both from Ghana, had genotypes consistent with mixed infection with A and D patterns (HU11020 and HU13281, Table S1). Massive parallel sequencing of the entire LMP-1 gene and promoter of these two biopsies revealed general heterozygosity of 25% in one sample and 55% in the other sample, but the heterogeneity at specific positions varied at individual positions—ranging from 6.5% to 99% (Table S1). Although it is theoretically possible that the apparently mixed infection may be due to ongoing mutational activity in the tumor leading to diversification EBV variants from D to A patterns [28], it is unlikely that such extensive genetic heterozygosity observed across the entire LMP-1 gene and promoter would be due to random mutations. A more likely explanation is that two independent viruses infected a single B cell that progressed to BL. We note that it is also possible that two independent cells with different infections progressed to BL and merged into one clinical tumor by colonizing the same tumor microenvironment. If so, examination of somatic mutations would reveal heterozygosity.

Our finding that LMP-1 Pattern A variants are enriched in EBV genomes sequenced from BL samples, and that variant Pattern A sub-variants are prevalent in BL, may be a clue that EBV in BL might be under selection pressure, and that the variations may be functionally relevant for tumor survival. 

### 2.4. Frequency of Pattern A in Peripheral Blood of 113 Children with- and 115 Children without-BL in Uganda

Table 5 shows the distribution of EBV LMP-1 patterns in peripheral blood of 113 children with eBL and 115 BL-free children enrolled from the same geographical areas as the cases in the EMBLEM study in Uganda. The samples were tested in two batches, starting with a proof-of-concept set of 13 children with BL and 15 BL-free children previously confirmed to be EBV positive using EBNA1 PCR [29]. These samples were tested by PCR using Lei LMP-1 primers [18], and the products sequenced by the Sanger method. Pattern A was observed in 15.4% (*n* = 2) of children with BL, but in 0% (based on *n* = 15) of the BL-free children. 

**Figure 5:**
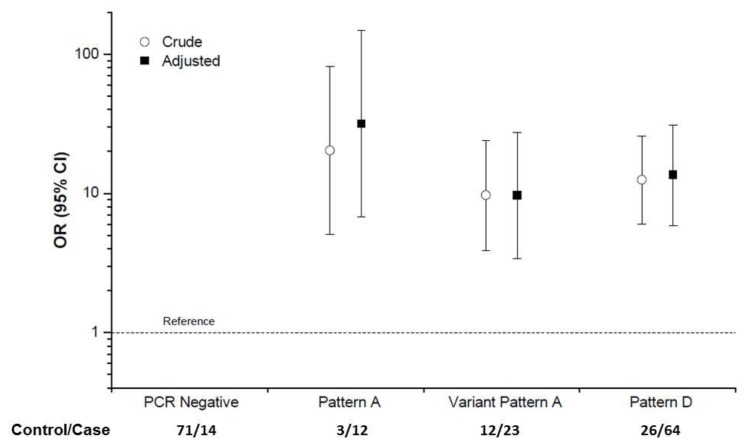
Association between LMP-1 patterns with Burkitt lymphoma in the EMBLEM Study in Uganda. The results are based on tests conducted in peripheral blood of 113 children with BL and 115 BL-free children recruited from the same geographical area as the cases. PCR negative children are used as the reference group with an odds ratio (OR) = 1, as indicated by the reference line.

Following the successful proof-of-concept experiment, the sample size was expanded to include peripheral blood from an additional set of 100 children with eBL and 100 BL-free children from the same geographical area. The combined results from the two sets are shown in Table 5. In this analysis, children who were PCR negative using Lei primers (see Methods Section 4.3) were used as the reference group, because negativity was interpreted as indicative of controlled EBV infection or absence of infection with the specific genotype. Pattern A variants were detected more frequently in peripheral blood of eBL cases than controls (10.6% versus 2.6%; Table 5). Pattern A variants were associated with a 20.3-fold (95% confidence interval [CI] 5.1–81.3) increase in the likelihood of eBL compared to PCR-negative subjects (Figure 5). We observed variant Pattern A sub-pattern variants in the peripheral blood of 20.3% eBL cases, compared to 10.4% of the controls in Uganda (Table 5). Pattern A sub-pattern variants were associated 9.7-fold (95% CI 3.9–24.0) odds for eBL risk compared to PCR-negative subjects. Pattern D was detected more frequently in eBL cases than in controls (56.6% versus 22.6%), and was associated with 12.5-times (95% CI 6.0–26.0) higher odds for eBL risk. Pattern B and Pattern C were not observed in these Ugandan samples.

This interpretation is reasonable because EBV infection in African children occurs during infancy [30], and EBV load is highest during the period of primary infection, and decreases significantly in chronically infected children [29,31]. Continued positivity and expression of virus at high levels is therefore an indication of poorly controlled viral infection and greater likelihood of EBV-mediated carcinogenesis [32]. This reasoning is similar to that commonly used for other virus-mediated cancers, notably cervical cancer, for which persistent infection with high-risk variants is considered the risk factor, and PCR negativity for high-risk types is used as the reference category [33].

Because detection of EBV may be related to shedding due to reactivation following infection with malaria [34], the associations with LMP-1 patterns were adjusted for malaria status (positive or negative), a history of malaria treatment as an inpatient or outpatient, rainfall season, and residence in a rural or urban village. The association between Pattern A with BL risk became stronger (OR 31.7), albeit with substantial uncertainty, whereas the ratio likelihood of associations with variant Pattern A sub-patterns or Pattern D were minimally altered, which suggests that those variants are not confounded by malaria-related variables. We note that the CIs for ORs for Pattern A, variant Pattern A, and Pattern D overlap, but this is not unexpected, and has been observed for associations between different HPV types and cervical cancer risk [33].

## 3. Discussion

### 3.1. LMP-1 Classification Using the Lei et al. Technique Applicable to HTS Data and Has Parsimony of Variants

We showed that LMP-1 genetic variants according to Lei et al. [18], Miller et al. [24], Hu et al. [23], and Edwards et al. [25] can be determined from HTS EBV data for the majority of cases. However, LMP-1 genetic diversity according to patterns reported by Sandvej et al. [27] can be determined from HTS data in less than 10% of cases. We observed that these different systems for classifying LMP-1 genetic diversity are independent of each other. This independence between classifications was illustrated by detailed comparisons of LMP-1 genetic diversity by Lei et al. [18] and by Edwards et al. [25], which showed that each Lei Pattern included 2–3 different Edwards strains, and vice versa. Thus, providing information using one system provides no information about the other system. The only exception was the Edward’s North Carolina strain [25], which was jointly classified only with Lei Pattern B [18]. Because our analysis suggests that Pattern B is predominant in people from the Far East, it is possible that the North Carolina strain also tracks with EBV from the Far East, although the finding that three Pattern B-North Carolina EBV samples were collected from individuals in Australia and an individual from Germany (all with unknown ancestry) suggests that this strain circulates elsewhere. The North Carolina strain was recently reported in about 12.2% of healthy donors from Qatar [35], but it was not found in a study of LMP-1 variants in the saliva and PBMCs in children in Argentina, who included people of Asian descent [36], suggesting that the North Carolina strain may not be specific for the EBV found in Far East populations. Taken together, our results provide a justification for conducting a secondary analysis of HTS data to evaluate LMP-1 variant-disease associations using these classifications.

### 3.2. Pattern A Variations Frequently Detected in BL Tumors or Peripheral Blood of eBL Cases

Our study was conducted to replicate our findings of enrichment of Pattern A in BL biopsies for a larger set of samples. We quadrupled the number of BL biopsies (from 14 to 64) that have been evaluated for Pattern A variations, and confirmed the high frequency (~50%) of Pattern A originally observed in BL biopsies from Ghana, Argentina, and Brazil [18]. In contrast, Pattern A is infrequently observed in healthy subjects, which supports the hypothesis that Pattern A may be a genetic marker of an EBV variant causal for eBL. Our lack of comparative data from BL-free patients from the same cities and towns as the BL patients from whom the biopsies were obtained is a limitation of our results, because the results in the BL cases may reflect geographical patterns of EBV diversity. In particular, EBV genetic patterns in Ghana and Brazil could be similar, because historical population movements between the two countries during the trans-Atlantic slave trade [37] may have led to similar EBV genetic variants circulating in the populations in the two countries. However, those reasons would not explain the similar frequency of the EBV Pattern A in BL cases in Argentina, which did not receive a large influx of Africans as Brazil. Our finding that Pattern A was infrequent in published EBV genomes in healthy people, as previously analyzed in our study [18], or those (*n* = 110 EBV genomes) published by Correia et al. [15], which we analyzed in the current study, suggests that Pattern A is indeed rare in healthy subjects, and is apparently enriched in BL EBV genomes. Further research is needed to investigate the frequency of LMP-1 Pattern A variants in healthy subjects in countries with a high eBL incidence.

### 3.3. Novel Pattern A-Like Variations Suggest a Much Broader Undiscovered LMP-1 Genetic Diversity

Our finding of novel variant Pattern A sub-variants, discovered during our replication study, suggests that the genetic diversity of the LMP-1 gene and promoter is not yet fully characterized. These results of sub-variants are not due to unconsciously modifying the definition of variants through post-hoc analysis, as we collected data from more BL samples or the peripheral blood samples in Uganda because we used the original definition of Pattern A [18]. These results from BL biopsies reflect the in vivo status of tumors’ individually sequenced DNA molecules, and do not reflect an ongoing mutational process that promotes variation within Pattern A during cell line propagation. Understanding the mechanisms that promote genomic diversity of EBV in tumors may help to elucidate the mechanism of nucleotide substitutions and their role in carcinogenesis. Our results encourage the study of EBV genetic diversity using HTS technology to accelerate the discovery of LMP-1 genetic diversity that may be associated with EBV-associated tumors. Further research using HTS data will provide a more comprehensive picture of EBV genetic diversity, and facilitate the discovery of variant-phenotype associations.

### 3.4. Pattern A and D Heterozygosity in Tumors Suggests Multiple EBV Infections in Single Pre-Cancer B Cells

Our finding that EBV in two BL biopsies from two subjects in Ghana had both Pattern A and D EBV strains is intriguing. Because tumors are presumed to arise from a single transformed cell, these data may be interpreted as suggesting that some tumors arise from a B cell infected with two different EBV strains, which has not been reported before [35,38,39]. This finding raises an interesting question about which viral sequence would be relevant for facilitating malignant transformation on one hand, and whether having both infections is a marker of specific individual vulnerability to EBV infection, on the other. We considered that the possibility that heterozygosity was due to contamination by a non-tumor virus was unlikely. The high proportion of heterozygosity of all positions in the LMP-1 gene was consistent with two viral strains being present in the tumor cells rather than isolated cells with a different virus contaminating the tumor. We considered the possibility that the EBV heterozygosity may be due to two independently infected B cells undergoing initiation and progression, and then fusing into one tumor. This hypothesis might be tested by investigating somatic changes in the tumor, which would be expected to reveal heterozygosity.

We note that accumulation of multiple substitutions in the tumor EBV leading to the generation of variant species of LMP-1 in vivo could contribute to EBV heterozygosity in tumors. Accumulation of multiple substitutions in the tumor EBV was reported in nasal T cell lymphomas from Peru [40], based on single strand conformational polymorphism (SSCP) analysis of EBNA-1 fragments from the tumor EBV, which was known to be monoclonal from analysis of EBV terminal repeat sequences. The multiple EBNA-1 variants were confirmed by direct sequencing of independently subcloned EBNA-1 PCR products in nasal T cell lymphomas [40]. If the accumulation of multiple substitutions contributes to EBV heterozygosity in BL, it will provide evidence for hypermutation as a process that generates quasi-species in EBV, with implications for study of tumor-specific variants and EBV vaccine research.

### 3.5. Implications of Detecting a High Frequency of Pattern A in BL Tumors or Peripheral Blood of eBL Cases

Our study focuses on LMP-1 genetic diversity as a field which is potentially useful for classifying EBV variants associated with elevated risk for BL. Our results from a larger series of BL biopsies confirmed the impression that LMP-1 Pattern A is enriched in EBV sequenced from BL biopsies. Our results from Uganda are the first study to demonstrate an association between LMP-1 Pattern A with eBL in cases compared to BL-free controls enrolled from the geographical area. The results showed a significantly elevated risk of eBL (OR 31.7) associated with detection of LMP-1 Pattern A in peripheral blood of the cases compared with the controls. However, an elevated risk of eBL with the detection of variant Pattern A sub-patterns and Pattern D was observed, albeit less strong than for Pattern A, suggesting that LMP-1 Pattern A is not the sole cause, although it is the strongest. 

The observation of variant Pattern A sub-patterns suggests that the genetic diversity of LMP-1 gene and promoter is yet to be fully characterized. A more comprehensive genetic description of this gene using full sequence data will clarify of the distribution of patterns, and facilitate the identification of specific SNVs that may be related to causal variants. Moreover, the detailed studies may provide insights about ongoing mutational processes and the mechanism for such genetic variation in tumor versus normal samples. 

Given the exciting associations noted above, our results are grounds for optimism; however, they are based upon the relatively small number of samples that have been studied to date. Thus, there is a need to increase the number of samples from healthy subjects from geographic areas where BL cases have been obtained to facilitate variant-phenotype studies. Secondly, there is a need to develop more sensitive assays to study variants, because the current PCR and Sanger sequencing assay targets large amplicons (435 bp, 428 bp, and 396 bp), and it is negative in up to 70% of peripheral blood samples in the controls. Failure to detect all infections could lead to detection bias in future studies. Because the relationship between LMP-1 patterns with EBV load is unknown, it is difficult to predict the direction of such bias, but it could attenuate findings to the null or bias findings away from the null. Thus, the development of more sensitive assays, coupled with an improvement in the number and geographical coverage of samples, would make it possible to conduct research to understand the relationship between LMP-1 patterns and viral load, to guide interpretation in future studies. Ideally, newer assays should seek to improve the assay detection limit of LMP-1 Patterns A in peripheral blood of healthy subjects, in order to enable precise evaluation of epidemiological associations between detection of LMP-1 Pattern A in body fluids and eBL risk.

We note that the current paradigm that LMP-1 is not expressed in EBV-positive BL tumors [41] has discouraged investigators from looking at genetic diversity in the LMP-1 gene in order to detect EBV high-risk variants. Even so, our results should encourage the study of LMP-1 biology in BL. LMP-1 is a CD 40 homolog [42,43] and may modulate a number of antigen-mediated B cell activation pathways [44]. The LMP-1 C-terminal activator region (CTAR) 1 and 2 modulate the expression of multiple anti-apoptotic proteins, and could influence lymphoma progression by suppressing cell death [45,46,47,48]. Also, LMP-1 may influence lymphomagenesis by modulating the production of cytokines, such as IL-6, IL-8, IL-10 [49], which regulate the expression of several B cell surface markers. However, our results should not be interpreted as proposing that LMP-1 is biologically involved in BL developed, but rather as a marker of the EBV variants associated with elevated risk for BL.

Our findings also highlight the need for cellular and animal studies to provide biological insights about the LMP-1 patterns reported here, and transform them from descriptive patterns to findings with biological significance for viral fitness, replication, transmission, and persistence. However, responding to the priorities of EBV research requires concerted cooperation between multi-disciplinary teams of scientists, in order to design studies that can be both epidemiologically rigorous and biologically informative.

## 4. Materials and Methods

### 4.1. Subjects and IRB

The fresh-frozen biopsies from patients with BL obtained mostly from the abdomen of affected children were collected from 71 subjects in South America (15 from Argentina, 11 from Brazil) and 45 from Ghana, and labeled with unique numbers in studies conducted by investigators at the National Cancer Institute between 1965 and 1994 [18]. Peripheral blood samples were collected from 113 children with, and 115 without, BL, all of whom were enrolled in the Epidemiology of Burkitt’s Lymphoma in East-African Children and Minors (EMBLEM) in Uganda between 2010 and 2016 (protocol 10-C-N133) [20]. Participation in the current study was approved by the Office of Human Research Protection at the National Institutes of Health. No identifying information was used in the current study.

### 4.2. DNA Extraction

The DNA extraction of the samples from South America and Ghana was described previously [50,51,52]. The DNA extraction of peripheral blood mononuclear cells (PBMCs) was processed at the NCI Cancer Genomics Research (CGR) Laboratory, Rockville, MD, USA, using the Qiagen QIAsymphony automated instrument [20].

### 4.3. PCR Amplification of the Targeted Hypervariable Region of LMP-1

The corresponding positions of the variants formed Pattern A, the PCR primer sets used for target amplification, and variant typing are shown in Figure S1 and Table S4. Three pairs of primers (Supplementary Figure S1) were used to cover the LMP-1 hypervariable region, containing 23 nucleotides (nt) variants. One hundred nanograms of DNA from BL tumor biopsy, and 300 ng DNA from peripheral blood mononuclear cells (PBMCs) were used as templates in a polymerase chain reaction (PCR). Each PCR mixture contained 2 µL 10× PCR buffer, 1.6 µL dNTPs (2.5 μM), 1 µL MgCl_2_ (10 mM), 0.5 µL primers (10 μM each), Platinum Taq DNA polymerase, and DNA template. Nuclease-free water was added to adjust the final volume. Thermocycle was carried out in Mastercycler Pro S (Eppendorf) (Eppendorf North America, Hauppauge, NY, USA) using initial denature at 94 °C for 5 min, thermocycle at 94 °C for 30 s, 55 °C or 60 °C for 30 s, and 72 °C for 30 s, total 45 cycles, following the final extension at 72 °C for 7 min. PCR products were separated by 2% agarose gel electrophoresis in 1×TAE buffer. The gel was stained with GelGreen (Biotium, Fremont, CA, USA) and the amplicons were visualized under blue light at wave length 460–520 µm (Amersham Imager 600, GE Healthcare, Marlborough, MA, USA).

We note that BL samples that were EBV negative were not subjected to Sanger sequencing. These included seven biopsies (3 from Argentina, one from Brazil and three from Ghana) and 85 peripheral blood samples from Uganda children (14 BL cases and 71 controls).

### 4.4. Sanger Sequencing and Data Analysis

The PCR generated amplicons matching the desired length were retrieved and eluted from the agarose gel using GeneClean III kit (MP Biomedicals, Santa Ana, CA, USA) and stored in RNase-free water. The purified amplicons from 64 BL biopsy samples and 143 peripheral blood samples were used as templates in the Sanger sequencing reactions, and the Sanger sequencing service was provided by Macrogen Inc. (Macrogen, Rockville, MD, USA). Three of the peripheral blood samples were unable to obtain a readable chromatogram. The exported chromatograms and sequence files were visualized and assembled against the type 1 EBV reference genome (NC_007605) using CLC Genomics Workbench Version 9.0.1. The sequence alignments were performed using BioEdit v7.0 with the ClustalW algorithm, and the sequences were classified manually. The bar charts were generated by the software package GraphPad Prism v7.0.

## 5. Conclusions

In conclusion, our analysis shows that HTS EBV data can be used to classify samples into classes of LMP-1 genetic diversity, as described by Lei et al. [18], Miller et al. [24], Hu et al. [23], and Edwards et al. [25], but not according Sandvej et al. [27]. Of these, LMP-1 Pattern A, based on Lei et al. [18], was frequently (55–80%) detected in BL biopsies from Ghana, Argentina, and Brazil, but less frequently in EBV genomes from healthy people (~10%). In a case-control analysis of peripheral blood samples from Uganda, compared to PCR-negative subjects, detection of LMP-1 Pattern A in peripheral blood was associated with a significantly elevated risk (OR = 31.7) of eBL. eBL risk was elevated, but lower than for variant Pattern A sub-variants (OR 9.7) or Pattern D (OR 13.6). These results support the hypothesis that Pattern A could be a marker for elevated risk for EBV variants associated with BL risk, although it is not the sole variant. Further research is needed to replicate and elucidate our findings in other settings.

## Figures and Tables

**Table 1:** Schematic representation of four unique patterns of variations (A to D) discovered in the Epstein-Barr virus latent membrane protein 1 (LMP-1) gene hypervariable region by Lei et al. [18]

Characteristic	LMP-2B Exon1													LMP-1 Promoter																											LMP-1 Exon1							LMP-1 Exon3			
**nt position**	1. −426	2. −412	3. −410	4. −367	−372	−356	5. −354	−329	−328	−315	−286	−284	−240	−238	−234	−233	6. −227	−207	−199	7. −184	8. −172	−163	−136	−70	9. −50	−44	−43	−37	−34	10. −39	−17	11. −12	−6	−4	−3	+11	+12	12. +18	+26	+32	+41	13. E2D	14.15. H3L	R17L	16. V43I	17. S57A	18.19. I63V	20. I124V	21. I152L	22. H213N	23. E214Q
**nt change**	G>A	T>G	C>A	G>A	G>A	C>A	A>G	C>T	G>A	C>T	A>G	G>T	G>A	A>G	G>T	G>A	G>A	C>T	C>T	A>T	T>C	C>T	A>T	A>C	T>A	G>T	A>T	C>G	C>G	A>C	A>T	G>A	C>T	C>A	C>G	A>G	C>T	T>G	A>C	G>A	G>C	GAA>GAC	CAC>CTG	CGA>CTA	GTT>ATT	TCC>GCC	ATA>GTG	ATC>GTC	ATC>CTC	CAT>AAT	GAA>CAA
**Pattern A** **(23 variants)**	A	G	A	A	G	C	G	C	G	C	A	G	G	A	G	C	A	C	C	T	C	C	A	A	A	C	A	C	C	C	A	A	C	C	C	A	C	G	A	G	C	C	TG	G	A	G	GG	G	C	A	C
**Pattern B** **(29 variants)**	G	T	C	G	A	A	A	T	A	T	G	T	A	G	T	A	G	T	T	A	T	T	T	C	T	C	T	G	G	A	T	G	T	A	G	G	T	T	C	A	G	A	AC	T	G	T	AA	A	A	C	G
**Pattern C** **(3 variants)**	G	T	C	G	G	C	A	C	G	C	A	G	G	A	G	G	G	C	C	A	T	C	A	A	T	T	A	C	C	A	A	G	C	C	C	A	C	T	A	G	C	C	AC	G	G	T	AA	A	A	C	G
**Pattern D ** **(wild-type)**	G	T	C	G	G	C	A	C	G	C	A	G	G	A	G	G	G	C	C	A	T	C	A	A	T	G	A	C	C	A	A	G	C	C	C	A	C	T	A	G	G	A	AC	G	G	T	AA	A	A	C	G

Abbreviations: nt nucleotide; Pattern A: 23 single nucleotide variants, including 11 variants in the promoter and 9 amino acid changes in the coding sequence of LMP-1. The single nucleotide variations used to define Pattern A are numbered for ease of reference to guide the definition of Pattern A in other datasets. Pattern B: 29 single nucleotide variants, including 28 variants in the promoter and 1 amino acid change in the coding sequence of LMP-1. Pattern C: 3 single nucleotide variants, including 2 variants in promoter and sharing amino acid change E2D with Pattern A. Pattern D: The LMP-1 wild type sequence of the EBV reference genome NC_007605. The colors indicate the specific nucleotides that vary (dark green for Pattern A, lime green for Pattern B, and blue for Pattern C); only nt changes in Pattern A are numbered.

**Table 2:** A comparative analysis of EBV the LMP-1 genetic diversity in 114 EBV genomes analyzed in the study of Lei et al. (2015) using four commonly used classifications compared to the classification proposed by Lei et al. [18]

EBV Genome	Lei et al. [18] Patterns	Source	Origin	EBV Type	Hu et al. [23] Loss of Xho I Site	Miller et al. [24] 30 bp Deletion	33 bp Repeats (^#^ of RP Unit)	^†^ Edwards et al. [25] C-Terminal Variants	^#^ Sandvej et al. [27] Group
KP968263**(H058015C)	A	BL biopsy	Ghana	Type 1	No	Yes	4	China 1	ND
KP968262**(H018436D)	A	BL biopsy	Ghana	Type 1	No	Yes	4	China 1	ND
KP968264**(H002213)	A	BL biopsy	Ghana	Type 1	No	Yes	4	China 1	ND
KP968261**(HU11393)	A	BL biopsy	Ghana	Type 1	No	Yes	4	ND	ND
KR063342**(H03753A)	A	BL biopsy	Ghana	Type 1	No	No	4	China 1	ND
KR063345**(FNR)	A	BL biopsy	Brazil	Type 1	No	Yes	4	China 1	ND
KR063344**(RPF)	A	BL biopsy	Brazil	Type 1	No	Yes	4	China 1	ND
KP968258**(MP)	A	BL biopsy	Brazil	Type 1	No	Yes	4	China 1	ND
KP968257**(CCH)	A	BL biopsy	Brazil	Type 1	No	Yes	4	China 1	ND
KR063343**(CV-ARG)	A	BL biopsy	Argentina	Type 1	No	Yes	4	China 1	ND
KT001102 (VA)	A	BL biopsy	Argentina	N/A	Gap	Gap	4	China 1	ND
KT001103 (SG)	A	BL biopsy	Argentina	Type 1	No	Gap	4	China 1	ND
KP968259**(SCL)	A	BL biopsy	Brazil	Type 1	No	No	4	B95–8	ND
LN827554(LCL-AFB1)	A	LCL	Unknown	Type 2	No	Yes	5	China 1	ND
LN824206(pLCL-TRL1-post)	A	sLCL. PTLD (post)	USA	Type 1	No	Yes	5	China 1	ND
LN824207(pLCL-TRL1-pre)	A	sLCL. PTLD (pre)	USA	Type 1	No	Yes	5	China 1	ND
LN827591(sLCL-2.15)	A	sLCL	Kenya	Type 2	No	Yes	5	China 1	ND
LN827594(sLCL-IS1.07)	A	sLCL. PTLD	Australia	Type 1	No	Yes	5	China 1	ND
LN827559(pLCL-TRL595)	A	sLCL. PTLD	USA	Type 1	No	Yes	5	China 1	ND
LN827563(sLCL-1.18)	A	sLCL	Kenya	Type 1	No	Yes	5	China 1	ND
KF717093**(Raji)	A	BL	Nigeria	Type 1	No	No	4	B95-8	ND
KP968260**(VGO)	B	BL biopsy	Brazil	Type 1	Yes	No	5	ND	ND
KC207813**(Akata)	B	BL	Japan	Type 1	Yes	Yes	4	China 1	ND
LN824208**(Akata)	B	BL	Japan	Type 1	Yes	Yes	4	China 1	ND
KC617875(C666-1)	B	NPC	Asia	Type 1	Yes	Yes	4	China 1	ND
KJ411974(C666-1)	B	NPC	Asia	Type 1	Yes	Yes	1	China 1	ND
KC617875(C666-1)	B	NPC	Asia	Type 1	Yes	Yes	4	China 1	ND
AY961628(GD1)	B	NPC	China	Type 1	Yes	Yes	4	China 1	ND
HQ020558(GD2)	B	NPC	China	Type 1	Yes	Yes	3	China 1	ND
KF373730(M81)	B	NPC	Asia	Type 1	Yes	Yes	4	China 1	ND
LN824142(Saliva)	B	Healthy saliva	UK	Type 1	Yes	Yes	2	China 1	ND
LN827562(sLCL-1.19)	B	sLCL	Kenya	Type 1	Yes	No	4	ND	ND
LN827561(YCCEL1)	B	GC cell line	South Korea	Type 1	Yes	Yes	5	China 1	ND
LN824209(HKN14)	B	NPC	Hong Kong	Type 1	Yes	Yes	5	China 1	ND
LN827547(HKN15)	B	NPC	Hong Kong	Type 1	Yes	Yes	4	China 1	ND
LN824224(HKN19)	B	NPC	Hong Kong	Type 1	Yes	Yes	4	China 1	ND
LN827549(D3201.2)	B	NPC	China	Type 1	Yes	Yes	6	China 1	ND
JQ009376(HKNPC1)	B	NPC	Hong Kong	Type 1	Yes	Yes	5	China 1	ND
KF992564(HKNPC2)	B	NPC	Hong Kong	Type 1	Yes	Yes	1	China 1	ND
KF992565(HKNPC3)	B	NPC	Hong Kong	Type 1	Yes	Yes	4	China 1	ND
KF992566(HKNPC4)	B	NPC	Hong Kong	Type 1	Gap	Gap	Gap	Gap	ND
KF992567(HKNPC5)	B	NPC	Hong Kong	Type 1	Yes	Yes	1	China 1	ND
KF992568(HKNPC6)	B	NPC	Hong Kong	Type 1	Yes	Yes	1	China 1	ND
KF992569(HKNPC7)	B	NPC	Hong Kong	Type 1	Yes	Yes	1	China 1	ND
KF992570(HKNPC8)	B	NPC	Hong Kong	Type 1	Yes	Yes	2	China 1	ND
KF992571(HKNPC9)	B	NPC	Hong Kong	Type 1	Yes	Yes	1	China 1	ND
LN827523(L591)	B	HL cell line	Germany	Type 1	Yes	No	4	NC	ND
LN827799(sLCL-IM1.16)	B	sLCL. IM.	Australia	Type 1	Yes	No	4	NC	ND
LN827578(sLCL-IS1.13)	B	sLCL. PTLD	Australia	Type 1	Yes	No	4	NC	ND
LN827586(sLCL-IS1.15)	B	sLCL. PTLD	Australia	Type 1	Yes	No	4	NC	ND
LN827800**(Jijoye)	C	BL	Nigeria	Type 2	No	No	4	B95-8	ND
LN827548**(P3HR1_c16)	C	BL	Nigeria	Type 2	No	No	4	B95-8	ND
LN827557**(BL36)	C	BL	N. Africa	Type 1	No	No	4	B95-8	ND
LN827545**(Daudi)	C	BL	Kenya	Type 1	No	No	4	Med	ND
LN827551**(Makau)	C	BL	Kenya	Type 1	No	No	5	Med	ND
LN824205(sLCL-1.12)	C	sLCL	Kenya	Type 1	No	No	5	Med	ND
LN824203**(Mak1)	C	BL	Kenya	Type 1	No	No	5	Med	ND
LN827544**(Wewak1)	D	BL	PNG	Type 2	No	No	4	ND	ND
LN827556**(Cheptages)	D	BL	Kenya	Type 2	No	No	4	B95-8	ND
LN827526**(BL37)	D	BL	Africa	Type 1	No	Yes	6	Med	ND
KC207814**(Mutu)	D	BL	Kenya	Type 1	No	No	4	Med	ND
NC_009334**(AG876)	D	BL	Ghana	Type 2	No	Yes	4	China 1	ND
NC_007605 (WT-EBV)	D	B98-8	USA	type 1	No	No	4	B95-8	ND
AJ507799(WT-EBV)	D	B98-8	USA	Type 1	No	No	4	B95-8	ND
V01555(WT-EBV)	D	B95-8	USA	Type 1	No	No	4	B95-8	ND
KC440851(K4123-Mi)	D	healthy donor	USA	Type 1	No	No	4	Med	ND
KC440852(K4413-Mi)	D	healthy donor	USA	Type 1	No	No	4	B95-8	**A**
NA19114	D	healthy donor	Yoruba	Type 1	No	No	4	B95-8	ND
NA19315	D	healthy donor	Kenya	Type 1	No	No	4	B95-8	ND
NA19384	D	healthy donor	Kenya	Type 1	Gap	No	4	China 1	ND
LN827739(LCL_B958)	D	LCL, B95-8	USA	Type 1	No	No	4	B95-8	ND
LN827597(sLCL-IS1.04)	D	sLCL. PTLD	Australia	Type 1	No	No	5	B95-8	ND
LN827596(sLCL-IM1.02)	D	sLCL. IM.	Australia	Type 1	No	Yes	6	China 1	ND
LN827595(sLCL-IS1.03)	D	sLCL. PTLD	Australia	Type 1	No	No	5	Med	ND
LN827593(sLCL-IS1.12)	D	sLCL. PTLD	Australia	Type 1	No	No	4	B95-8	**A**
LN827592(sLCL-IS1.10)	D	sLCL. PTLD	Australia	Type 1	No	No	5	Med	ND
LN827590(sLCL-IM1.05)	D	sLCL. IM.	Australia	Type 1	No	Yes	6	China 1	ND
LN827589(sLCL-IS2.01)	D	sLCL. PTLD	Australia	Type 2	No	Yes	4	China 1	ND
LN827588(sLCL-IS1.19)	D	sLCL. PTLD	Australia	Type 1	No	No	5	B95-8	**A**
LN827587(sLCL-2.21)	D	sLCL	Kenya	Type 2	No	Yes	4	China 1	ND
LN827585(sLCL-1.04)	D	sLCL	Kenya	Type 1	No	No	4	Med	ND
LN827584(sLCL-IS1.06)	D	sLCL. PTLD	Australia	Type 1	No	No	4	Med	ND
LN827583(sLCL-IM1.17)	D	sLCL. IM.	Australia	Type 1	No	No	4	B95-8	ND
LN827582(sLCL-BL1.03)	D	sLCL	Kenya	Type 1	No	No	4	B95-8	ND
LN827581(sLCL-1.05)	D	sLCL	Kenya	Type 1	No	No	4	Med	ND
LN827580(sLCL-2.16)	D	sLCL	Kenya	Type 2	No	No	5	B95-8	ND
LN827579(sLCL-1.13)	D	sLCL	Kenya	Type 1	No	No	4	Med	ND
LN827577(sLCL-1.17)	D	sLCL	Kenya	Type 1	No	No	4	Med	**A**
LN827576(sLCL-IS1.20)	D	sLCL. PTLD	Australia	Type 1	No	Yes	7	China 1	ND
LN827575(sLCL-IS1.14)	D	sLCL. PTLD	Australia	Type 1	No	No	4	B95-8	**A**
LN827574(sLCL-1.09)	D	sLCL	Kenya	Type 1	No	No	4	Med	**A**
LN827573(sLCL-1.10)	D	sLCL	Kenya	Type 1	No	No	3	Med	ND
LN827572(sLCL-IS1.18)	D	sLCL. PTLD	Australia	Type 1	No	No	4	B95-8	ND
LN827571(sLCL-BL1.20)	D	sLCL	Kenya	Type 1	No	No	4	Med	ND
LN827570(sLCL-IS1.01)	D	sLCL. PTLD	Australia	Type 1	No	Yes	4	China 1	ND
LN827569(sLCL-IS1.11)	D	sLCL. PTLD	Australia	Type 1	No	No	5	Med	ND
LN827568(sLCL-1.24)	D	sLCL	Kenya	Type 1	No	No	4	Med	ND
LN827567(sLCL-IM1.09)	D	sLCL. IM.	Australia	Type 1	No	No	4	B95-8	ND
LN827566(sLCL-1.06)	D	sLCL	Kenya	Type 1	No	No	4	Med	ND
LN827565(sLCL-1.07)	D	sLCL	Kenya	Type 1	No	No	4	Med	ND
LN827564(HL04)	D	HL	UK	Type 1	No	Yes	3	China 1	ND
LN827560(sLCL-2.14)	D	sLCL	Kenya	Type 2	No	Yes	4	China 1	ND
LN827558(sLCL-1.02)	D	sLCL	Kenya	Type 1	No	No	4	Med	ND
LN827555(X50-7)	D	LCL	USA	Type 1	No	No	4	B95-8	ND
LN827553(sLCL-IS1.08)	D	sLCL. PTLD	Australia	Type 1	No	No	5	Med	ND
LN827552(sLCL-1.08)	D	sLCL	Kenya	Type 1	No	No	4	Med	ND
LN827550(sLCL-1.11)	D	sLCL	Kenya	Type 1	No	No	4	Med	ND
LN827546(HL02)	D	HL	UK	Type 1	No	Yes	3	China 1	ND
LN827527(M-ABA)	D	LCL, NPC virus	N. Africa	Type 1	No	No	4	B95-8	**A**
LN827524(HL11)	D	HL	UK	Type 1	No	No	5	Med	ND
LN827522(HL09)	D	HL	UK	Type 1	No	Yes	6	China 1	ND
LN824226(HL01)	D	HL	UK	Type 1	No	Yes	3	China 1	ND
LN824225(HL08)	D	HL	UK	Type 1	No	Yes	5	China 1	ND
LN824204(HL05)	D	HL	UK	Type 1	No	Yes	5	China 1	ND

Notes: List sorted by LMP-1 Patterns according to Lei et al. [18] and then by phenotype (BL, LCL, NPC, and healthy donor). No: means the variant is absent, Yes: means the variant is present, Gap: means the sequence was ambiguous or gapped, therefore, the variant cannot be called. ^†^ Some of the C-terminal variants used for classification in the study of Edwards et al. (1999) were found in the 114 EBV genomes [25]. The samples were assigned following the classifications (B95-8, China 1, North Carolina (NC), Mediterranean (Med)) used in the study of Edwards et al. (1999) [25] # Sandvej [27] classification made the LMP-1 variants into 4 groups, Group A: 6 SNV on the coding sequence (CDS), containing neither loss of Xho I site nor 30 bp deletion, Group B: 33 bp repeats different from which of the B95-8 plus 19 SNVs on CDS, Group C: 30 bp deletion plus 44 SNVs on LMP-1 CDS, Group D: loss of Xho I sit plus 66 SNVs in the LMP-1 CDS and 33 SNVs in the promoter. Because the number of the repeat units is not accurate when the sequence is coming from shotgun sequencing or HTS, the estimated number is not good for classification; thus, many samples cannot be classified. These samples are designated not determined (ND) to indicate that the classification is not applicable.

**Table 3:** Table showing novel LMP-1 Pattern A-like sub-variations discovered in samples evaluated by PCR-Sanger method.

Gene Region		LMP-2B Exon1								LMP-1 Promoter				LMP-1 Exon1								LMP-1 Exon3			
Primer pair		Lei-F3/Lei-R3								Lei-F1/Lei-R1												Lei-F2/Lei-R2			
Amplicon size		396 bp								435 bp												428 bp			
**FT and a.a. affected**							AML1	LBF2	LBF4		CREB			E2D	H3L		S24A (new)	V43I	S57A	I63V		I124V	I152L	H213N	E214Q
**Variation**		1. G-426A	2. T-412G	3. C-410A	4. G-376A	5. A-354G	6. G-227A	7. A-184T	8. T-172C	9. T-50A	10. A-39C	11. G-12A	12. T+18G	13. GAA>GAC	14. CAC>CTG	15. CAC>CTG	TCC>GCC	16. GTT>ATT	17. TCC>GCC	18. ATA>GTG	19. ATA>GTG	20. ATC>GTC	21. ATC>CTC	22. CAT>AAT	23. GAA>CAA
**Pattern**	**Example**																								
**A**	**BLS000073**	A	G	A	A	G	A	T	C	A	C	A	G	C	T	G	T	A	G	G	G	G	C	A	C
**Mid-Length A**	**BLS000207**	G	T	C	G	A	G	A	T	T	A	A	G	C	T	G	T	A	G	G	G	G	C	A	C
**4nt sub-pattern A**	**BLS000292**	G	T	C	G	A	G	A	T	T	C	G	T	C	T	G	G	G	T	A	A	A	A	C	G
**3nt sub-pattern A**	**BLS000020**	G	T	C	G	A	G	A	T	T	A	G	T	C	T	G	G	G	T	A	A	A	A	C	G
**Partial A sub-pattern**	**BLS001943**	A	G	A	A	G	A	T	C	A	C	A	G	C	T	G	T	G	T	A	A	A	A	C	C

Abbreviations: nt nucleotide; Notes: The colors indicate the specific nucleotides that vary (light green show nucleotide changes in Mid-length A; blue shows 4 nt sub-pattern A; grey show 3 nt sub-pattern A; light orange shows partial A sub-pattern A).

**Table 4:** Frequency of the EBV LMP-1 patterns in EBV directly sequenced from BL biopsies in Latin America and Ghana.

Geographical Region	PCR Positive *	PCR Negative *	Pattern A	Mid Length-A	Pattern D	Pattern B	Pattern C	Pattern A/D
**Argentina**	15	3	8 (66.7%)	2 (16.7%)	2 (16.7%)	0 (0%)	0 (0%)	0 (0%)
**Brazil**	11	1	8 (80.0%)	0 (0%)	1 (10.0%)	1 (10.0%)	0 (0%)	0 (0%)
**Ghana**	45	3	23 (54.8%)	5 (11.9%)	11 (26.2%)	0 (0%)	1 (2.4%)	2 (4.8%)
**Total (N)**	71	7	39 (60.9%)	7 (10.9%)	14 (21.9%)	1 (1.6%)	1 (1.6%)	2 (3.1%)

* PCR performed using Lei Primers as described in Lei et al. [18]. Only PCR positive samples were subject to Sanger sequencing.

**Table 5:** Frequency of the LMP-1 patterns in EBV in peripheral blood samples in 143 children with or without BL in the EMBLEM Study in Uganda evaluated by PRC and Sanger Sequencing.

Sample Batch	Group	Number of PBMCs	PCR Positive *	PCR Negative	Pattern A	Mid-Length A sub-Pattern	Partial A sub-Pattern	4nt sub-Pattern A	3nt sub-Pattern A	Pattern D	N.D. ^#^
**Batch 1**	**BL cases**	13	13 (100%)	0 (0%)	2 (15.4%)	0 (0%)	0 (0%)	1 (7.7%)	1 (7.7%)	9 (69.2%)	0 (0%)
	**Controls**	15	15 (100%)	0 (0%)	0 (0%)	4 (26.7%)	0 (0%)	4 (26.7%)	0 (0%)	7 (46.7%)	0 (0%)
**Batch 2**	**BL cases**	100	86 (86%)	14 (14%)	10 (10.0%)	7 (7.0%)	3 (3.5%)	8 (9.3%)	3 (3.5%)	55 (55.0%)	0 (0%)
	**Controls**	100	29 (86%)	71 (71%)	3 (3.0%)	4(4.0%)	0 (0%)	0 (0%)	0 (0%)	19 (19.0%)	3 (3.0%)
**Batch 1 and 2**	**BL cases**	113	99 (87.6%)	14 (12.4%)	12 (10.6%)	7 (6.2%)	3 (2.6%)	9 (8.0%)	4 (3.5%)	64 (56.4%)	0 (0%)
	**Controls**	115	41 (35.6%)	71 (64.4%)	3 (2.6%)	8 (7.0%)	0 (0%)	4 (3.5%)	0 (0%)	26 (22.6%)	3 (2.6%)

Abbreviations: PBMCs peripheral blood mononuclear cells. Percent are calculated considering all the samples, which are shown in the column headed “Number of PBMCs”. For association analyses (Figure 5), Mid-Length A, Partial A, 4nt- and 3nt variants are combined into one group called “Variant Pattern A”. The association of LMP-1 variants are compared to PCR negative as the reference group, similar to the practice in the HPV field (see Munoz N et al. [33]). * The PCR positivity of both batches was tested using Lei-1 primers (see methods). ^#^ N.D.: Undetermined samples were PCR-positive, but the product was insufficient for Sanger sequencing.

## Supplementary Materials

The following are available online at http://www.mdpi.com/2072-6694/10/6/177/s1, Figure S1: The illustration of the corresponding positions of the 23 single nucleotide variants that form the pattern A on LMP-1 gene and the primers used for the variant typing via target-specific PCR and Sanger sequencing. Table S1: Heterogeneous variations in the LMP-1 gene suggestive of co-infection with Pattern A and Pattern D EBV in 2 BL tumors from Ghana. Table S2: EBV LMP-1 Patterns in 22 EBV-positive BL tumors from South America typed using PCR amplicon sequencing via Sanger method. Table S3: EBV LMP-1 Patterns in 42 EBV positive BL tumors from Ghana typed using PCR amplicon sequencing via Sanger method. Table S4: The primers were used for PCR amplifying the hypervariable region of the LMP-1 and Sanger Sequencing. Table S5: The summary of the Lei et al. pattern classification applied on the samples from BL cases and healthy donors that had been used for LMP-1 phylogenic analysis in the study of Correia et al. (2017). Table S6: The geographic distribution and the Pattern A-D classification of the BL cases and healthy donors that were used in the study of Correia et al. (2017).

## Abbreviations

The following abbreviations are used in this manuscript:

EBV	Epstein-Barr virus
LMP-1	Latent membrane protein-1
EBNA-1	Epstein–Barr nuclear antigens-1
BZLF-1	Trans-activator protein BZLF1
HTS	High throughput sequencing
SNV	Single nucleotide polymorphism
PCR	Polymerase chain reaction
BL	Burkitt lymphoma
eBL	endemic Burkitt lymphoma
NPC	Nasopharyngeal carcinoma
PTLD	Post-transplant lymphoproliferative disease
HL	Hodgkin Lymphoma
GC	EBV-positive gastric carcinoma
IM	Infectious mononucleosis
EMBLEM	Epidemiology of Burkitt’s Lymphoma in East-African Children and Minors
sLCL	Spontaneous transformed lymphoblastoid cell line
CTAR-1, 2, 3	Carboxyl-terminal activator regions 1, -2, -3
IL-6, -8, -10	Interleukin-6, -8, -10
SSCP	Single strand conformational polymorphism
