# Supplementary material for: Frequency of EBV LMP-1 Promoter and Coding Variations in Burkitt Lymphoma Samples in Africa and South America and Peripheral Blood in Uganda
Source: Cancers (Basel). 2018 Jun 2;10(6):177. doi: 10.3390/cancers10060177 (PMC6024959; doi:10.3390/cancers10060177)
Supplement: Supplementary file 1 [file cancers-10-00177-s001.pdf]

Supplementary Materials: Frequency of EBV LMP-1 Promoter and Coding Variations in Burkitt Lymphoma Samples in Africa and South America and Peripheral Blood in Uganda

Hsiao-Mei Liao, Hebing Liu, Heiyan Lei, Bingjie Li, Pei-Ju Chin, Shien Tsai, Kishor Bhatia, Marina Gutierrez, Sidnei Epelman, Robert J Biggar, Francis Nkrumah, Janet Neequaye, Martin D. Ogwang, Steven J. Reynolds, Shyh-Ching Lo and Sam M. Mbulaiteye

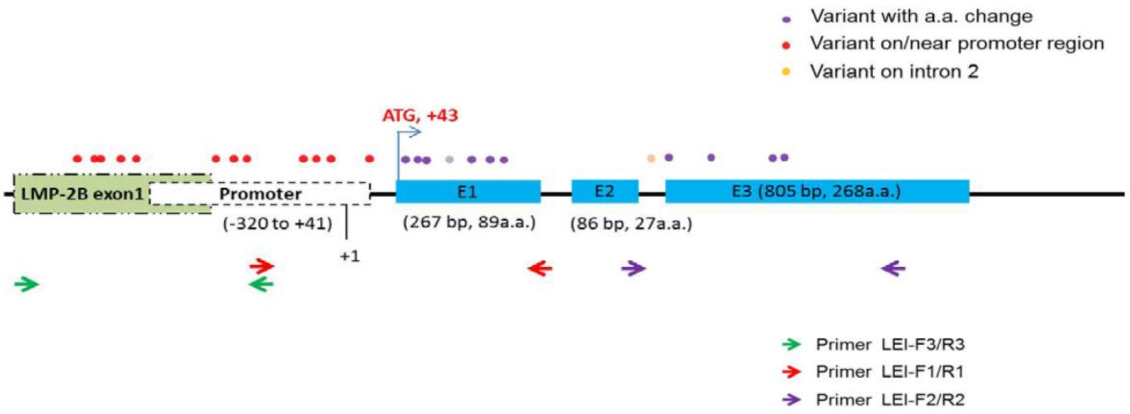

Figure S1: The illustration of the corresponding positions of the 23 single nucleotide variants that form the pattern A on LMP-1 gene and the primers used for the variant typing via target-specific PCR and Sanger sequencing

Table S1: Heterogeneous variations in the LMP-1 gene suggestive of co-infection with Pattern A and Pattern D EBV in 2 BL tumors from Ghana.

		**Gene region**	**LMP-2B exon 1**					**LMP-1 promoter**							**LMP-1 exon1**								**LMP-1 exon3**			
		**Primer pair**	**Lei-F3/ Lei-R3**								**Lei-F1/ Lei-R1**												**Lei-F2/ Lei-R2**			
		**Amplicon size**	**396 bp**								**435 bp**												**428 bp**			
		**FT and a.a. affected**						AML1	LBF2	LBF4		CREB			E2D	H3L		S24A (new)	V43I	S57A	I63V		I124V	I152L	H213N	E214Q
		**Variation**	1. G-426A	2. T-412G	3. C-410A	4. G-376A	5. A-354G	6. G-227A	7. A-184T	8. T-172C	9. T-50A	10. A-39C	11. G-12A	12. T+18G	13. GAA>GAC	14 CAC>CTG	15. CAC>CTG	TCC>GCC	16. GTT>ATT	17. TCC>GCC	18. ATA>GTG	19. ATA>GTG	20. ATC>GTC	21. ATC>CTC	22. CAT>AAT	23. GAA>CAA
**Origin**	**Pattern**	**Sample**																								
Ghana	**A/D**	**HU11020**	G/A	T/G	C/A	G/A	A/G	G	T	T/C	T/A	A/C	G/A	T/G	A/C	A/T	C/G	T	G/A	T/G	A/G	A/G	A/G	A/G	C/A	G/C
	**Heterozygosity (%)**		56.0	54.4	54.2	53.3	56.4	0	98.7	52.8	50.6	54.1	55.9	55.4	79.4	55.3	55.3	0	56.8	57.1	57.8	57.8	49.6	49.6	49.4	49.5
	**A/D**	**HU13281**	G/A	T/G	C/A	G/A	G	G	A/T	T/C	T	A	A	T	C	A	C	T	G	T	A	A	A/G	A/C	C/A	G/C
	**Heterozygosity (%)**		24.8	24.8	24.6	25.3	99.8	0	24.6	24.8	0	0	99.5	0	99.7	0	0	0	0	0	0	0	7.3	7.5	6.5	6.5

Table S2: EBV LMP-1 Patterns in 22 EBV-positive BL tumors from South America typed using PCR amplicon sequencing via Sanger method.

Sample Name	Geographic distribution	EBV detection		Variant typing result	
		Southern Blot	PCR	LMP-1 Pattern	Typing method
CV	Argentina	+	+	A	HTS[#]/PCR-Sanger
VA	Argentina	+	+	A	
SG	Argentina	+	+	A	
N=3					
MD	Argentina	+	+	A	PCR-Sanger
JR	Argentina	-	+	A	
BD	Argentina	-	+	A	
RJR	Argentina	-	+	A	
135	Argentina	-	+	A	
HD	Argentina	-	+	ML-A	
AF	Argentina	+	+	ML-A	
OJI	Argentina	+	+	D	
AA	Argentina	+	+	D	
AS	Argentina	-	-	N.D.	
AG	Argentina	-	-	N.D.	
RJ	Argentina	-	-	N.D.	
N=12					
FNR	Brazil	+	+	A	HTS[#]/PCR-Sanger
CCH	Brazil	+	+	A	
RPF	Brazil	+	+	A	
MP	Brazil	+	+	A	
SCL	Brazil	+	+	A	
VGO	Brazil	+	+	B	
N=6					
SRC	Brazil	-	+	A	PCR-Sanger
IN	Brazil	-	+	A	
BE	Brazil	-	+	A	
ND	Brazil	-	+	D	
ME	Brazil	-	-	N.D.	
N=5					

Table S3: EBV LMP-1 Patterns in 42 EBV positive BL tumors from Ghana typed using PCR amplicon sequencing via Sanger method.

Sample name	Geographic distribution	EBV Detection		Variant typing result	
		Southern Blot	PCR	LMP-1 Pattern	Typing Method
H058015C	Ghana	+	+	A	HTS#/PCR-Sanger
HU11393	Ghana	+	+	A	
H018436D	Ghana	+	+	A	
H002213	Ghana	+	+	A	
H03753A	Ghana	+	+	A	
N= 5					
H02212D	Ghana		+	A	PCR-Sanger (N=40)
HU11541	Ghana		+	A	
HU11595	Ghana		+	A	
HU12335	Ghana		+	A	
HU11308	Ghana		+	A	
HU12208	Ghana		+	A	
HU11394	Ghana		+	A	
HU10416	Ghana		+	A	
HU10377	Ghana		+	A	
HU13148	Ghana		+	A	
HU11813	Ghana		+	A	
H057519a	Ghana		+	A	
H058001a	Ghana		+	A	
H057520a	Ghana		+	A	
H057521a	Ghana		+	A	
H003840a	Ghana		+	A	
HU13312	Ghana		+	A	
HU14473	Ghana		+	A	
N= 18					
HU14163	Ghana		+	ML-A	
HU11386	Ghana		+	ML-A	
HU14250	Ghana		+	ML-A	
HU10615	Ghana		+	ML-A	
HU10612	Ghana		+	ML-A	
N= 5					
HU13265	Ghana		+	C	
N= 1					
HU10552	Ghana		+	D	
HU11303	Ghana		+	D	

HU11469	Ghana		+	D	
HU11344	Ghana		+	D	
HU10496	Ghana		+	D	
HU11361	Ghana		+	D	
HU11589	Ghana		+	D	
HU14219	Ghana		+	D	
HU10244	Ghana		+	D	
H057518a	Ghana		+	D	
H031201b	Ghana		+	D	
N= 11					
HU12078	Ghana		-	N.D.	
HU12317	Ghana		-	N.D.	
HU14423	Ghana		-	N.D.	
N= 3					
HU11020	Ghana		+	Mix A/D	
HU13281	Ghana		+	Mix A/D	
N= 2					
	Total N=45				

Notes: These samples include 5 that previously were typed using whole genome high-throughput sequencing
#: The partial genome and the addressed LMP-1 variant pattern has been published by Lei, H., et al. on *Sci Rep* **5**: 16706 (2015).
N.D.: undetected

ML-A: 12 or 13 serial variants (variants number 11 to 23 or 12 to 23 on table 1) of the 23 Pattern A variants presented in the sample were assigned as Middle Length pattern A.

Table S4: The primers were used for PCR amplifying the hypervariable region of the LMP-1 and Sanger Sequencing.

**Primer Name**	**Sequence 5′ to 3′**	**Amplicon Length (bp)**	**Tm (°C)**
LEI-1F	GCCTCCGGCAGACCCCGCAAATC	435	55
LEI-1R	GGGCAAAGGGTGTAATACTTAC		
LEI-2F	CCTTGGAATTGTGCTGTTCA	428	55
LEI-2R	TCTGCCCTCGTTGGAGTTAG		
LEI-3F	GTCTGCTGCTTCGTCACCCGCCGAC	396	60
LEI-3F	GATTTGCGGGGTCTGCCGGAGGC		

Table S5: The summary of the Lei et al. pattern classification applied on the samples from BL cases and healthy donors that had been used for LMP-1 phylogenic analysis in the study of Correia et al. (2017). Table S6: The geographic distribution and the Pattern A-D classification of the BL cases and healthy donors that were used in the study of Correia et al. (2017).

	**BL tumor biopsy (N=3)**	**BL Cell line (N=16)**	**Saliva from BL patient (N=4)**		**Total BL cases (N=23)**	**healthy saliva, sLCL (N= 59)**
**Pattern A**	3 (Argentina)	1 (Africa)	0	N	4	0
				%	17.4%	0.0%
**Pattern B**	0	2 (Africa)	0	N	2	30
				%	8.7%	50.8%
**Pattern C**	0		1 (Africa)	N	1	3
				%	4.3%	5.1%
**Pattern D**	0	13 (Africa)	3 (Africa)	N	16	26
				%	69.6%	44.1%

Note: In the Correia et al. study, total sample number for LMP-1 phylogenic analysis was 190. Eighty of the 190 samples were overlapped in the Lei et al. study. Rests of the 110 samples were subjected to the pattern classification. The classification was based on the amino acid sequences of each sample obtained from the Supplementary table S2 of the Correia study. The nucleotide sequences of the LMP-1 promoter region of these samples are unavailable. The samples from BL cases and saliva or sLCL from healthy donors of the Correia et al. study was described in this table. The samples from other disease states in the same study were not included.

© 2018 by the authors. Submitted for possible open access publication under the terms and conditions of the Creative Commons Attribution (CC BY) license (http://creativecommons.org/licenses/by/4.0/).
